# Congestion Control in CoAP Observe Group Communication

**DOI:** 10.3390/s19153433

**Published:** 2019-08-05

**Authors:** Chanwit Suwannapong, Chatchai Khunboa

**Affiliations:** Department of Computer Engineering, Faculty of Engineering, Khon Kaen University, 40002 Khon Kaen, Thailand

**Keywords:** Internet of Things, constrained application protocol, wireless sensor networks, congestion control, observing resource, group communication

## Abstract

The Constrained Application Protocol (CoAP) is a simple and lightweight machine-to-machine (M2M) protocol for constrained devices for use in lossy networks which offers a small memory capacity and limited processing. Designed and developed by the Internet Engineering Task Force (IETF), it functions as an application layer protocol and benefits from reliable delivery and simple congestion control. It is implemented for request/response message exchanges over the User Datagram Protocol (UDP) to support the Internet of Things (IoT). CoAP also provides a basic congestion control mechanism. In dealing with its own congestion, it relies on a fixed interval retransmission timeout (RTO) and binary exponential backoff (BEB). However, the default CoAP congestion control is considered to be unable to effectively perform group communication and observe resources, and it cannot handle rapid, frequent requests. This results in buffer overflow and packet loss. To overcome these problems, we proposed a new congestion control mechanism for CoAP Observe Group Communication, namely Congestion Control Random Early Detection (CoCo-RED), consisting of (1) determining and calculating an RTO timer, (2) a Revised Random Early Detection (RevRED) algorithm which has recently been developed and primarily based on the buffer management of TCP congestion control, and (3) a Fibonacci Pre-Increment Backoff (FPB) algorithm which waits for backoff time prior to retransmission. All the aforementioned algorithms were therefore implemented instead of the default CoAP mechanism. In this study, evaluations were carried out regarding the efficiency of the developed CoCo-RED using a Cooja simulator. The congestion control mechanism can quickly handle the changing behaviors of network communication, and thus it prevents the buffer overflow that leads to congestions. The results of our experiments indicate that CoCo-RED can control congestion more effectively than the default CoAP in every condition.

## 1. Introduction

The Internet of Things (IoT) is a technology which has been developed to provide internet connectivity in various networking systems, everyday objects, and electronic devices. Such connectivity works under the IEEE 802.15.4 standard [1] and relies on IP-based systems which employ IP for communication; e.g., Thread and 6LoWPAN. To account for such communication, the Internet Engineering Task Force (IETF) [2] developed a Constrained Application Protocol (CoAP), which is a specialized web transfer protocol for constrained devices and constrained networks. Its operations are similar to those of a Hypertext Transfer Protocol (HTTP) [3]. In addition, the CoAP runs over the User Datagram Protocol (UDP) instead of the Transmission Control Protocol (TCP) and does not require a three-way handshake and the maintenance of a connection status; it is therefore considered to be more suitable for constrained devices and technological operations that support communications between objects or devices or so-called machine-to-machine (M2M) protocols. A commonplace example can be taken from an application that helps monitor and send warnings/alerts about natural disasters. This application generates continuous traffic and burst traffic. Sensor nodes in a network tend to receive or cache sets of captured sensory data together and then deliver them to a base station, resulting in a huge load of information in the network [4,5,6,7,8].

Generally, there are two communication patterns, both CoAP-based and Observing Resource (Obs), which are considered as one-to-one communication [9]. Since a variety of application domains require data from IoT devices, data communication between a sender and a receiver is essential, contributing to the emergence of group communication [10,11,12,13]. At the same time, congestion control for group communication has also been developed as a crucial communication platform for networks with a large number of sensor nodes [14,15,16,17,18]. Under these circumstances, only the congestion control for CoAP-based Group Communication, or the so-called default CoAP mechanism, has been developed. This functions similarly to TCP congestion control, but it is based on the CoAP message exchange via UDP by a request message (a confirmable message) which is transferred from a client to a server. This also requires the client to wait for an acknowledgement message with the data/response that the client needs. The default CoAP mechanism is, however, not suitable for Obs congestion control. When notifications are sent from the server to the client, the system users are unable to determine the duration of the delay before each notification, and they cannot determine the data rate for such notifications. These problems have led to the development of the congestion control mechanism for CoAP in order to facilitate the observation of group communication.

In this article, we introduce the Congestion Control Random Early Detection (CoCo-RED) mechanism which combines the use of the RevRED algorithm for buffer management and the FPB algorithm to obtain retransmission timeout (RTO) estimations for the transmission of the CoAP messages. CoCo-RED was designed to deliver a congestion control that is not only adaptive to network conditions but suitable for IoT characteristics.

The rest of the paper is organized as follows. In Section 2, we summarize how the CoAP yields congestion control mechanisms and then derive two core mechanisms from it. We also analyze how these two core mechanisms are implemented in the CoAP. In Section 3, we propose a new approach to a congestion control mechanism for CoAP Observe Group Communication: CoCo-RED. In Section 4, we introduce a simulation setup and communication protocol stack configurations that can be used to carry out performance evaluations of CoCo-RED and compare them to the default CoAP mechanism. The results of these evaluations are presented in Section 5, and the conclusions of this paper are provided in Section 6.

## 2. CoAP Congestion Control

CoAP is regarded as a Representational State Transfer style (RESTful) protocol [19] designed for constrained nodes and constrained networks which support IoT functions. With lightweight messages, it requires fewer resources and less server energy consumption than other competing protocols. It can also support activations from clients. As mentioned earlier, it was designed and developed by the Internet Engineering Task Force (IETF) [20]. It falls under a two-layer structure: (1) a messaging layer which deals with the UDP for communications, and (2) a request/response layer which supports the RESTful protocol. The latter is based on the URI/URL of the request message for retrieving data.

Broadly speaking, CoAP defines four message or transaction types: (1) confirmable (CON), (2) non-confirmable (NON), (3) acknowledgement (ACK), and (4) reset (RST) [19]. Regarding the CoAP message exchange in the request/response layer interaction model, the request layer deals with either the CON request or the NON request in terms of message transmission. The response layer deals with delivering messages by means of a piggyback response which goes along with the ACK message. In other words, reliable message transmission in the CoAP occurs due to the CON message, which is reliable and is based on the default timeout and Binary Exponential Backoff (BEB) during the retransmissions. At this stage, the server that receives the data sends back the ACK message with exactly the same (confirmable) message ID (MID) as the client previously delivered. On the other hand, for unreliable message transmissions, the CoAP can send the NON message, meaning the server does not send back the ACK response. The server itself records the MID to detect the same message instead.

**Figure 1:**
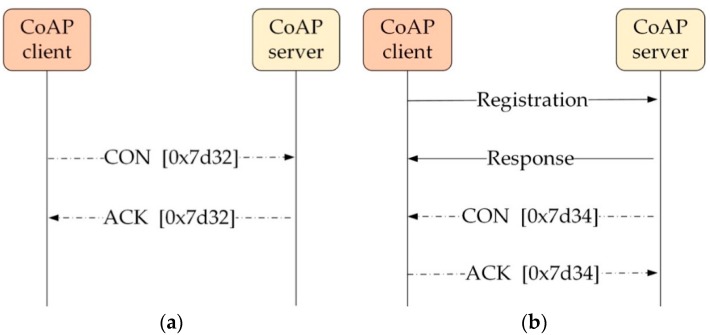
(**a**) Constrained Application Protocol (CoAP)-based message transmission; (**b**) Observing Resource message transmission. CON: confirmable; ACK: acknowledgement.

Specifically, the reliable message transmission [21,22,23] of the CoAP for the CON message exchange is classified into two types: (1) the CoAP (CoAP-based) protocol, and the Observing Resource (Obs) protocol, as illustrated in Figure 1a,b, respectively. It is notable that the Obs, which this study focuses on, requires registration from the client as an observer. Then, the server sends notifications with the CON message. This differs from the CoAP-based protocol whereby the CON message is initially sent from the client.

**Figure 2:**
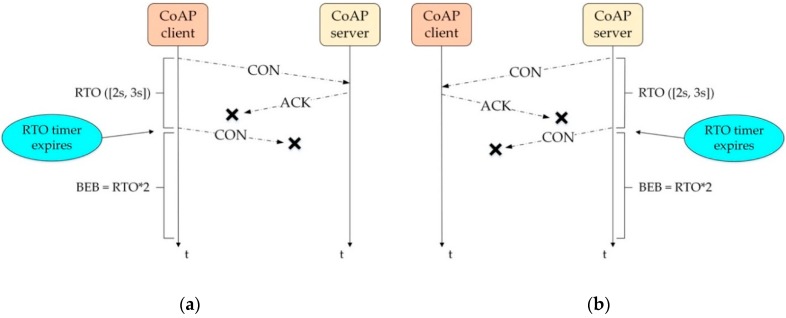
(**a**) Default CoAP-CON mode; (**b**) Observing Resource-CON mode. RTO: retransmission timeout; BEB: binary exponential backoff.

Another important aspect relies on the CoAP congestion control [17,18], which was established to limit the delay between requests for message transmission and to limit the number of messages exchanged. As the CoAP works on the four message types previously mentioned, this operation is performed under the request/response layer. The reliable messages in the CoAP-based communication from either the CoAP-based transmission or the Obs transmission were obtained using the CON message and required an ACK message from the server. In addition, the congestion control mechanisms allow the CON message to retransmit up to four times before considering the transmitted message to be an error or failure. For the first message transmission, the RTO value was randomly selected from an interval of 2–3 s as an initial value. The BEB was also applied to the RTO, causing the RTO value for each transmission to double. This was, therefore, similar to TCP [3]. Please see Figure 2 for the whole picture of the congestion control via reliable messages.

The default CoAP mechanism typically relies on RTO and BEB as its two core algorithms. Indeed, the CoAP does not adjust the RTO in accordance with the round-trip time (RTT). Consequently, if the chosen RTO value for the default CoAP mechanism is below a certain RTT, this may result in a failure of retransmission from the CoAP. On the other hand, the CoAP tends to be implemented in networks where the bit error rate (BER) is high, and it can produce long idle times if the RTO value greatly overestimates the RTT.

## 3. CoCo-RED: Congestion Control Random Early Detection

According to the problems of the CoAP congestion control mechanisms suggested in the previous section, we propose the Congestion Control Random Early Detection (CoCo-RED) mechanism, which emerges from Random Early Detection (RED) gateways [24] for congestion avoidance in packet-switched networks. However, from a standard linear model of the packet drop function, it was found that the packet drop always occurred when the average queue size (*AvgQ*) was higher than the maximum threshold [25]. Thus, all the packets were dropped continuously, which might have some negative effects regarding the efficiency of a network. In this study, we adjusted this operation using an exponential function to lower the drop probability, but this can also cause the buffer to overflow; therefore, consecutive packets would be dropped. Such problems can be addressed by signaling sources to slow down the packet drop at the initial stage, followed by increasing the rate of the packet drop if the queue size is close to the size of the buffer. Our proposed CoCo-RED mechanism was comprised of three major components: an RTO calculation randomly selected from the interval of 2–4 s, buffer management from the client, and retransmission. All the components were under the control of this mechanism in both the client and the server, which is explained in the next paragraph.

**Figure 3:**
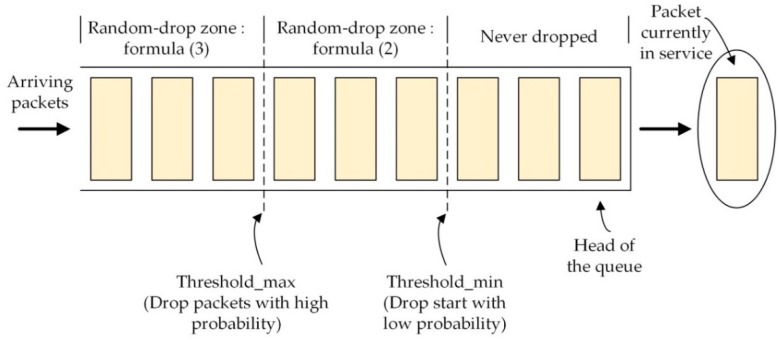
Congestion Control Random Early Detection (CoCo-RED) thresholds in the buffer queue.

**Figure 4:**
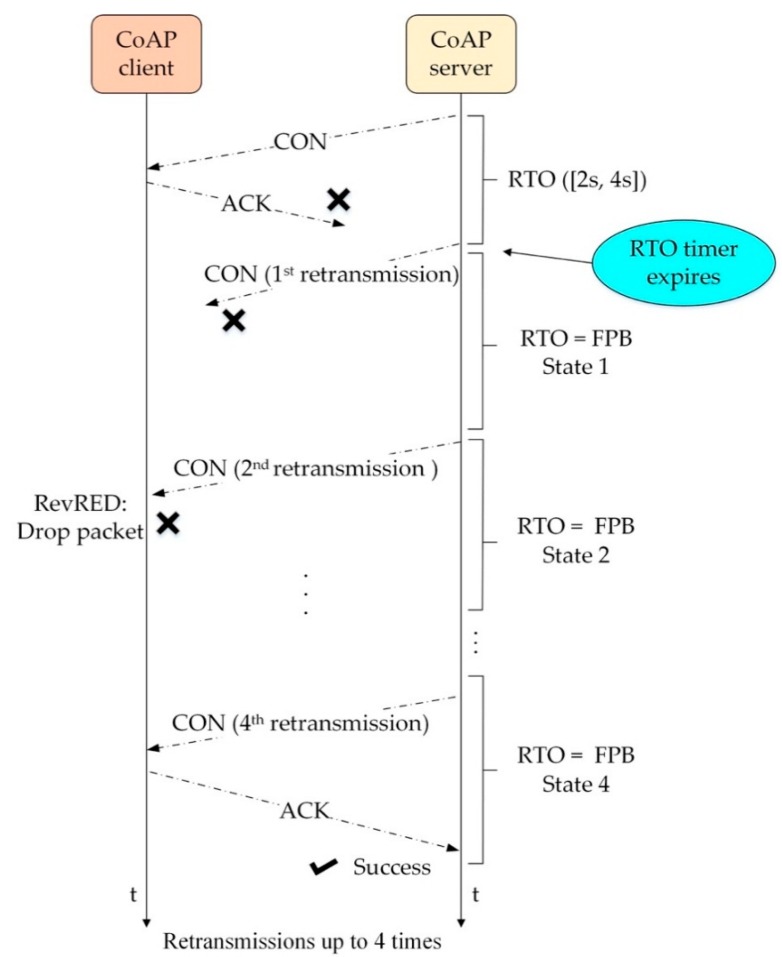
CoCo-RED timing diagram. FPB: Fibonacci Pre-Increment Backoff.

Regarding the outstanding feature of our CoCo-RED mechanism, it works dynamically, depending on the Revised Random Early Detection algorithm (RevRED), which calculates network density from the *AvgQ*. To illustrate this, as network congestion starts, the RevRED can solve this problem by dropping arriving packets in the system before the queue in the client’s buffer overflows. Owing to statistical probabilities corresponding to *AvgQ* that prevent burst traffic congestion, the buffer queue management of the RevRED algorithm is shown in Figure 3. Another distinctive feature of this mechanism is that when a packet drop occurs in the RevRED algorithm, there is a backoff for the first retransmission or until the packet which had the same previous MID can be sent or acknowledged successfully. The backoff time can be calculated with a Fibonacci Pre-Increment Backoff (FPB) algorithm. The waiting period for retransmission helps to reduce network traffic since the sever nodes at which the packets were dropped cannot communicate. Also, this indirectly causes the queue on the client’s buffer to decrease. Figure 4 shows the timing diagram of CoCo-RED and the functions of the CoCo-RED mechanism. The figure is subsequently explained in two parts: the server and the client.

**Figure 5:**
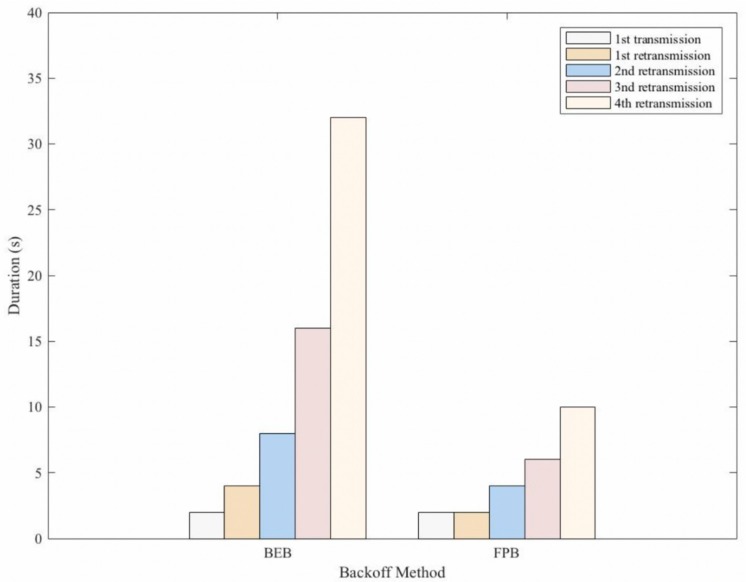
Backoff method durations for one message transmission and four message retransmissions, starting with *RTO_init_* = 2 s.

- Server: The server starts calculating and determining the RTO timer by randomly selecting in intervals of 2–4 s. Then, it sends the CON message to the client, and the server needs an ACK message response within the period of the RTO timer; otherwise, the server undergoes retransmission. In this manner, the CoCo-RED mechanism allows retransmissions up to four times before considering the notification an error or failure, which was similar to the default CoAP. Moreover, in each retransmission, the FPB (i.e., Fibonacci fib n, n = {1, 2, 3, 5}) was multiplied with the *RTO_previous_* to determine the new RTO timer for the next retransmission. The work of FPB is shown in Algorithm 1, and the duration of each retransmission is illustrated in Figure 5. Figure 5 compares the backoff durations for one message transmission and four message retransmissions in the BEB and the FPB, starting with the *RTO_init_* value of 2 s. Notably, the total duration before timeout was 62 s for the BEB and 24 s for the FPB. This means that the fast retransmission of FPB contributes to flexible buffer management and rapid response times.
**Algorithm 1.** Fibonacci Pre-Increment BackoffInitialize random value from [2 s, 4 s] to *RTO_init_*Initialize Fibonacci to [1, 2, 3, 5]when transmitting CON   RTO = *RTO_init_*   **for**
*i* = 0 to (size of Fibonacci)-1      *RTO_previous_* = RTO      **if** RTO expires without having received an ACK = RTO       RTO = *RTO_previous_* * Fibonacci[*i*]       *i* = * i* + 1      **else**   return transmission success return transmission fail**endfor**
- Client: The client works based on the RevRED algorithm, which is illustrated in Algorithm 2. When the client has obtained the CON message from the Server, it calculates the *AvgQ* via the exponential weighted moving average (EWMA) [26], which can be seen in Formula (1) (below). This operation conforms to three principles:
  (1) If the *AvgQ* is lower than *Threshold_min_,* the incoming packet is accepted or placed in the queue.
  (2) If the *AvgQ* is between *Threshold_min_* and *Threshold_max_*, the incoming packet is dropped in accordance with the dropping probability in Formula 2. Then, the client waits for a notification with the same MID which arises from the server’s retransmission.
  (3) If the *AvgQ* is equal to or beyond *Threshold_max_*, the incoming packet is dropped in accordance with the exponential dropping probability in Formula 3. Similar to the second principle, the client waits for a notification with the same MID which arises from the server’s retransmission.

In Formula (2) to Formula (3), dropping the packet when the *AvgQ* in the buffer is greater than the threshold can help to decrease the traffic congestion in a particular network and lessen the communication burdens on the client’s side. Thus, when the packet is dropped, the server still waits for the backoff during the retransmission before resending the notification.
**Algorithm 2.** Revised Random Early Detection (RevRED)for each packet arrival calculate the average queue size *AvgQ* (Formula 1) **if**
*Threshold_min_* < *AvgQ* < *Threshold_max_*    calculate probability *p_d_(AvgQ)* (Formula 2)    with probability *p_d_(AvgQ)*: drop packet **else if**
*Threshold_max_* < *AvgQ* ≤ maximum buffer    calculate probability *p_d_(AvgQ)* (Formula 3)    with probability *p_d_(AvgQ)*: drop packet

(1) $$AvgQ\;\leftarrow \;(1-w_{q})\;AvgQ+w_{q}q$$

(2) $$p_{d}(AvgQ)=\frac{AvgQ-Threshold_{min}}{Threshold_{max}-Threshold_{min}}\times {max}_{p}$$

From the original exponential formula:

$$y=ab^{x}$$

where the limit of the buffer size is *K* and the max probability is 1 in order of the *(x,y)* value a and value b is expressed as

$$a=\frac{{max}_{p}}{{\left(e^{\frac{ln\;(1/{max}_{p})}{K-Threshold_{max}}}\right)}^{Threshold_{max}}}$$

$$b=(e^{\frac{ln\;(1/{max}_{p})}{K-Threshold_{max}}}).$$

Therefore, for given *a* and *b* values, the actual probability of an arrived packet being dropped is computed as

(3) $$p_{d}(AvgQ)=\frac{{max}_{p}}{{\left(e^{\frac{ln\;(1/{max}_{p})}{K-Threshold_{max}}}\right)}^{Threshold_{max}}}\cdot {\left(e^{\frac{ln\;(1/{max}_{p})}{K-Threshold_{max}}}\right)}^{AvgQ}$$

**Figure 6:**
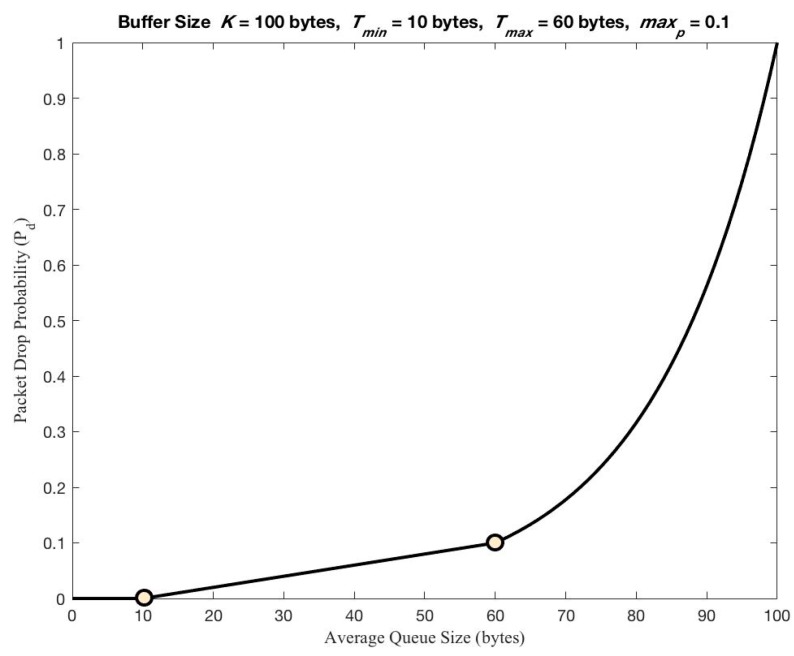
Packet drop probability function for CoCo-RED.

**Figure 7:**
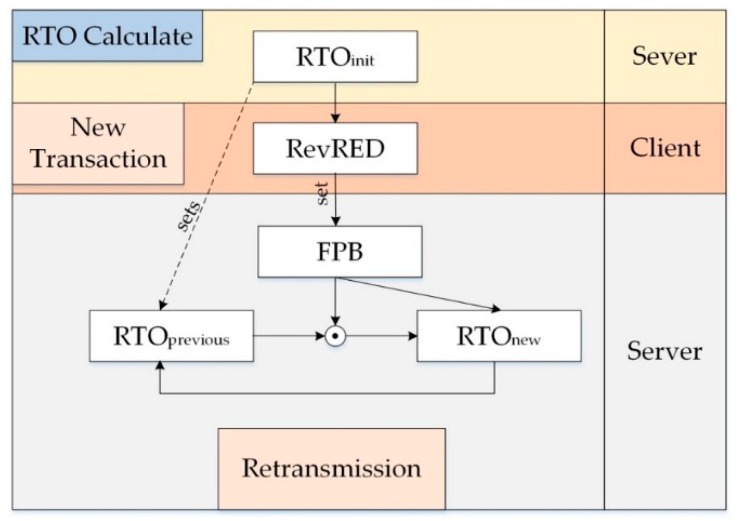
An overview of the RTO used to maintain and update the RTO state information for a destination endpoint in CoCo-RED.

In this regard, the *AvgQ* is the average queue length, *q* is the current queue size, *w_q_* represents the exponentially weighted moving average (equal to 0.002), and *K* denotes the limit of the buffer size. Figure 6 shows the packet drop probability function for CoCo-RED supposing that *K* = 100 bytes, *max_p_* = 0.1, *Threshold_min_* = 10 bytes, and *Threshold_max_* = 60 bytes. Figure 7 exhibits an overview of the RTO used to maintain and update the RTO state information for a destination endpoint in CoCo-RED.

## 4. Evaluation Setup

This section provides the details of evaluating the two congestion control mechanisms of default CoAP and CoCo-RED. This includes the simulator setup, the traffic scenarios, the network topologies and the performance metrics used to carry out the performance evaluations.

### 4.1. Simulation Setup

To simulate the network and evaluate our congestion control mechanism, the algorithms were implemented using the Cooja Simulation [27] in the Contiki Operating System (ContikiOS), an open-source operating system for constrained devices that supports dynamic loading and the replacement of individual programs and services [28]. The advantage of Cooja was that the program can compile and upload into a real node.

In this study, we selected a Z1 mote which was developed by Zolertia to be the principal node for simulations in the Cooja [29]. It was devised for a low-power wireless sensor network (WSN) which can communicate over the 6LoWPAN protocol. The hardware specifications of the Z1 mote are provided in Table 1, and the details of the Cooja parameter setup are in Table 2.

### 4.2. Traffic Scenarios

Two traffic scenarios were set to investigate the effects of the two congestion control mechanisms on the performance of the Obs communications.

- Continuous traffic: The CoAP server delivered the CON request notifications to the CoAP client. As the server obtained a reply from the client, it immediately transferred another CON request. Sending messages back-to-back from a large number of servers at the same time can create congestion. In this study, we used nine servers in order to achieve different levels of congestion. The test was performed continuously for 300 s and was repeated 20 times for each specific configuration.
- Burst traffic: This scenario simulated the burst traffic by increasing the congestion level in every topology. It started with a low congestion level at the server, where four nodes generated the continuous traffic (Obs) of back-to-back CON requests. Afterwards, a burst of traffic was generated by another group of servers consisting of the rest of the servers in the topology. For instance, network topology I had a group of three servers for burst traffic, i.e., three nodes, in order to achieve different levels of congestion. The test in this scenario was done in a similar manner to the continuous traffic scenario.

### 4.3. Network Topologies

**Figure 8:**
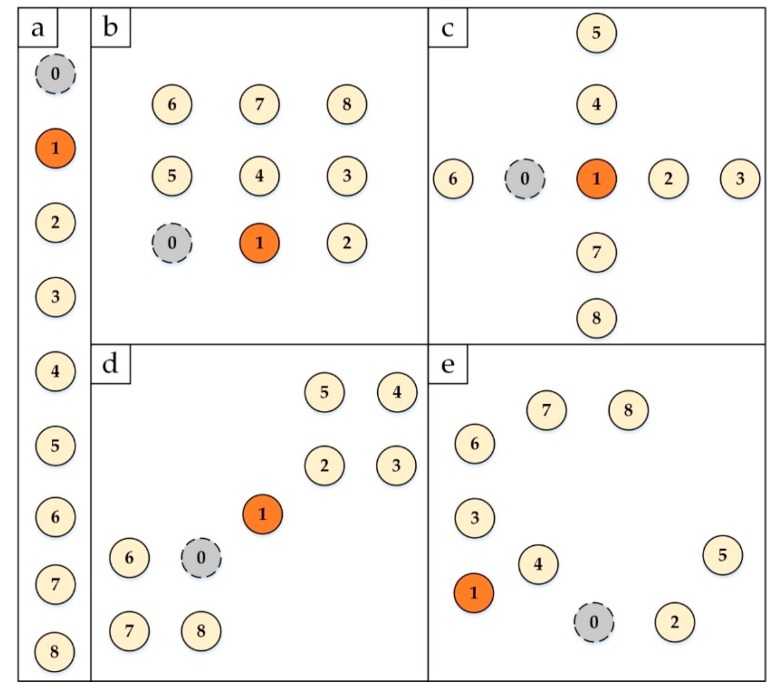
The five network topologies used for performance analysis (chain, grid, cross, dumbbell and random). The distance between the neighboring nodes is 10 m. The gray nodes are the RPL border routers. The orange nodes are the clients and the gold nodes are the groups of servers for the Obs messages.

In evaluating the efficiency of both of the CoAP congestion control mechanisms, the default CoAP and CoCo-RED, five different simulation network topologies were set up to conduct experiments in each scenario. Each topology differed in terms of the number of nodes, installation patterns, and hop counts for delivering the CoAP request from the source node to the destination node. The topologies used for the performance analysis were (a) a chain topology with 9 nodes, (b) a grid of 9 nodes (3 × 3), (c) a cross of 9 nodes, (d) a dumbbell topology with 9 nodes and (e) a random topology of 9 nodes. The number of CoAP nodes and the two-dimensional view of the node installation in different topologies is illustrated in Figure 8. The figure portrays the positions for installing the CoAP nodes in the five topologies. Each topology was conditioned to have a 10-m distance between nodes. The transmission range was 15 m while the interference range was 30 m. Therefore, in the chain topology, each node can select direct neighbors within the transmission range only, whereas in the other topologies, a node can have more than one neighbor.

Due to the limitations of the memory capacity within the Z1 platform operated in the Cooja simulator, several problems occurred when we increased the number of nodes, the network density, and the congestion levels. In future, we would like to test CoCo-Red using more complicated networks.

### 4.4. Performance Metrics

In Obs, appropriate performance metrics were chosen to evaluate the congestion control mechanism, concerning features of message transmission and congestion mechanisms on the CoAP. These metrics were designed to allow the measurement of the efficiency of the mechanisms as well as the performance in the network. They involved a simulation setup, traffic scenarios, and network typologies which were set under varying congestion levels for the test.

In the continuous traffic scenario and burst traffic scenario, the setting time, response time and packet loss were chosen as the important performance metrics since it was assumed that the CoAP transactions varied as a result of the changing topologies. Consequently, this would provide the whole picture of changes in the congestion level and the network size and help us to analyze the behaviors of the mechanism in each scenario.

## 5. Performance Evaluation

In this section, regarding the Obs, we compared the performance of our CoCo-RED with the default CoAP under the same continuous traffic scenario and burst traffic scenario and then provided a discussion of our evaluations of these mechanisms. We hypothesized that our developed CoCo-RED could control congestion more effectively with low packet loss and response time, yielding better network reliability and application support in group communication.

### 5.1. Continuous Traffic

In the continuous traffic scenario, the experiment aimed to explore the efficiency of the congestion control mechanism under typical network conditions. The messages were exchanged continuously by means of the Obs in the five topologies which determined different levels of congestion. The results were recorded following the performance metrics suggested in Section 4.4. Table 3 shows the overall performance metric values in the continuous traffic scenario for different topologies in the default CoAP, compared to those of CoCo-RED. Two metrics, hop count and setting time, served as the indicators for the congestion levels. The dumbbell topology resulted in the highest level of congestion, followed by the cross, the grid, the chain, and the random (as each node only selected direct neighbors within the transmission range in the chain topology, the messages were then exchanged in a unidirectional manner, and the setting time was eventually low in this study).

The response time and packet loss are also important performance metrics, especially for applications that require immediate reactions, short notification periods, and data integrity. The results from the two metrics in all network topologies suggest that CoCo-RED can handle congestion better than the default CoAP for both low congestion levels (i.e., the chain and random topologies) and high congestion levels (i.e., the grid, cross and dumbbell topologies). This was because, in CoCo-RED, the buffer management helped to measure the congestion levels of the network with the AvgQ controlling the packet drop rate prior to buffer overflow. Additionally, the retransmission timeout from FPB was significantly shorter than the BEB used by the default CoAP.

Overall, according to the performance metric values for the two mechanisms, CoCo-RED achieved a better performance than the default CoAP. Moreover, CoCo-RED can, in particular, effectively deal with high congestion levels in a network. This aligned with the aforementioned experiment on the packet loss and the response time. To summarize, CoCo-RED seems to be a smart choice for a congestion control mechanism in the Obs or in message exchanges in CoAP Group Communication which require quick responses under continuous traffic in a network.

### 5.2. Burst Traffic

In the burst traffic scenario, an experiment was conducted to investigate the efficiency of the default CoAP and CoCo-RED in five network topologies. The CoAP Observe Group Communication for the network with the burst traffic condition caused some difficulties in terms of data burst, according to this experiment. At the initial stage, we had set a typical communication environment with a small amount of message exchanges. Subsequently, the data input for message exchanges was so high in the network that it caused data burst and packet losses. The results in this section are in Table 4.

The results show that, in every network typology, the default CoAP was greatly affected by the simulated network communication, which can be supported by the evidence from such parameters as setting time, response time, and packet loss. When compared to the continuous traffic scenario in the previous section, these parameters turned out to be higher since the default CoAP congestion mechanism was only limited to the BEB algorithm and the RTO for each retransmission. This resulted in a higher response time and packet losses due to high congestion levels, as opposed to CoCo-RED, which was not significantly affected. This was because employing CoCo-RED can help to adjust the packet drop probability rate to be higher depending on the queue size in the buffer. It was able, therefore, to reduce the chances of buffer overflow during the burst traffic in the experiment. Likewise, the FPB algorithm was implemented to help determine the waiting time for the ACK messages from the group of servers. As regards the retransmission process, CoCo-RED did not have to wait for the messages to be resent, which led to shorter response times and fewer packet losses compared to the default CoAP.

The evaluation results of CoCo-RED compared to the default CoAP in the two traffic scenarios and the five topologies showed that CoCo-RED can effectively deal with congestion. With regard to this, CoCo-RED can, however, predict and reduce congestion by means of the packet drop and the FPB waiting time for the retransmission. We concluded that CoCo-RED can maintain the quality of within-network communication effectively.

## 6. Conclusions

In this article, we proposed a new congestion control mechanism for CoAP Observe Group Communication, namely CoCo-RED (Congestion Control Random Early Detection), based on buffer management and backoff algorithms in order to reduce the congestion of transmitted packets. Adapted from the TCP protocol, this congestion control mechanism can replace the previous CoAP. It can detect and predict congestion before packet loss occurs, especially when high network traffic density causes buffer overflow. Three major issues were also raised in this study: (1) RTO timer calculation, (2) average queue (AvgQ) and packet drop calculation, and (3) the use of the backoff algorithm for retransmission. The evaluation of the efficiency of CoCo-RED and the existing mechanism was performed using the Cooja simulator using two scenarios: the continuous traffic scenario and the burst traffic scenario. The experiment demonstrates that the CoCo-RED mechanism can help to predict and reduce the congestion from consecutive dropped packets in the case of buffer overflow. The efficiency of buffer management was higher when dealing with certain congestion levels. It can also help to reduce data traffic in the network to mitigate congestion, employing a wait for the backoff time until the timeout so that the retransmission occurs later. This, finally, reduces the packet loss and response time of network communication.

## Figures and Tables

**Table 1:** Hardware specification of the Zolertia Z1 wireless sensor node.

Mote	RAM	ROM	MCU	Radio
Zolertia (Z1)	8 KB	92 KB	MSP430F2167	CC2420

**Table 2:** Cooja parameter setup.

Settings	Value
Congestion mechanisms	Default CoAP, CoCo-RED
Routing protocol	Routing Protocol for Low-Power and Lossy Networks (RPL)
Max retransmissions	4
Wireless channel model	Unit Disk Graph model, transmission range = 15 m, interference range = 30 m
Distances between nodes	10 m
Transport and network	UDP + uIPv6 + 6LoWPAN
Media access control	(CSMA/CA)
Radio duty cycling (RDC)	Null-RDC
Radio band	2.4 GHz
Physical	IEEE 802.15.4 PHY
Simulation time	300 s
Max open transactions	8

**Table 3:** Overall performance metric values in a continuous traffic scenario for different topologies (the better-performing mechanism is highlighted in bold).

Topology	Congestion Control Mechanisms	Average Setting Time (s)	Average Response Time (s)	Average Packet Loss (s)
Grid	Default CoAP	17.74	0.67	2.92
	CoCo-RED	**14.18**	**0.59**	**1.63**
Chain	Default CoAP	13.91	0.65	1.30
	CoCo-RED	**13.26**	**0.54**	**1.00**
Cross	Default CoAP	18.09	0.74	3.31
	CoCo-RED	**17.78**	**0.70**	**3.07**
Dumbbell	Default CoAP	18.97	0.78	4.11
	CoCo-RED	**18.70**	**0.73**	**3.64**
Random	Default CoAP	13.26	0.55	-
	CoCo-RED	**12.77**	**0.44**	-

**Table 4:** Overall performance metric values in burst traffic scenario for different topologies (the better-performing mechanism is highlighted in bold).

Topology	Congestion Control Mechanisms	Average Setting Time (s)	Average Response Time (s)	Average Packet Loss (s)
Grid	Default CoAP	49.75	0.78	7.24
	CoCo-RED	**48.56**	**0.73**	**6.63**
Chain	Default CoAP	47.60	0.71	6.12
	CoCo-RED	**46.76**	**0.66**	**5.87**
Cross	Default CoAP	50.93	0.84	9.41
	CoCo-RED	**49.97**	**0.76**	**7.12**
Dumbbell	Default CoAP	51.48	0.92	10.64
	CoCo-RED	**50.81**	**0.84**	**7.58**
Random	Default CoAP	46.57	0.69	4.05
	CoCo-RED	**45.40**	**0.64**	**2.41**
