# Correction: Suwannapong, C. et al. Congestion Control in CoAP Observe Group Communication. *Sensors* 2019*, 19*, 3433

**DOI:** 10.3390/s19204387

**Published:** 2019-10-11

**Authors:** Chanwit Suwannapong, Chatchai Khunboa

**Affiliations:** Department of Computer Engineering, Faculty of Engineering, Khon Kaen University, 40002 Khon Kaen, Thailand; chanwit.s@kkumail.com

The authors wish to make the following corrections to this paper [1]:

We have found two inadvertent errors in our paper published in this Sensors [1]:

1. In Section 3. of this paper [1], the sentence “Moreover, in each retransmission, the FPB (i.e., Fibonacci fib n, n = {1, 2, 3, 5}) was multiplied by the *RTO_previous_* to determine the new RTO timer for the next retransmission.” This sentence should be modified to state: “Moreover, in each retransmission, the FPB (i.e., Fibonacci fib n, n = {1, 2, 3, 5}) was multiplied by the *RTO_init_* to determine the new RTO timer for the next retransmission.”

2. In Section 3. of this paper [1], the following Algorithm 1 should be replaced with the Algorithm shown below it.
**Algorithm 1.** Fibonacci Pre-Increment Backoff Initialize random value from [2 s, 4 s] to *RTO_init_* Initialize Fibonacci to [1, 2, 3, 5] when transmitting CON RTO = *RTO_init_* **for**
*i* = 0 to (size of Fibonacci)-1   *RTO_previous_* = RTO   **if** RTO expires without having received an ACK = RTO     RTO = *RTO_previous_* * Fibonacci[*i*]     *i* = *i*+1   **else** return transmission success return transmission fail **endfor**
**Algorithm 1.** Fibonacci Pre-Increment Backoff Initialize random value from [2 s, 4 s] to *RTO_init_* Initialize Fibonacci to [1, 2, 3, 5] when transmitting CON RTO = *RTO_init_* **for**
*i* = 0 to (size of Fibonacci)-1   **if** RTO expires without having received an ACK     RTO = *RTO_init_* * Fibonacci[*i*]     *i* = *i*+1    **else**     return transmission success  **endfor** return transmission fail

The changes do not affect the scientific results. The manuscript will be updated, and the original will remain online on the article webpage, with a reference to this Correction. The authors would like to apologize for any inconvenience caused to the readers by these changes.
